# Supplementary figures and images for: Transcriptome analysis reveals increased abundance and diversity of opportunistic fungal pathogens in nasopharyngeal tract of COVID-19 patients
Source: PLoS One. 2023 Jan 19;18(1):e0278134. doi: 10.1371/journal.pone.0278134 (PMC9851516; doi:10.1371/journal.pone.0278134)

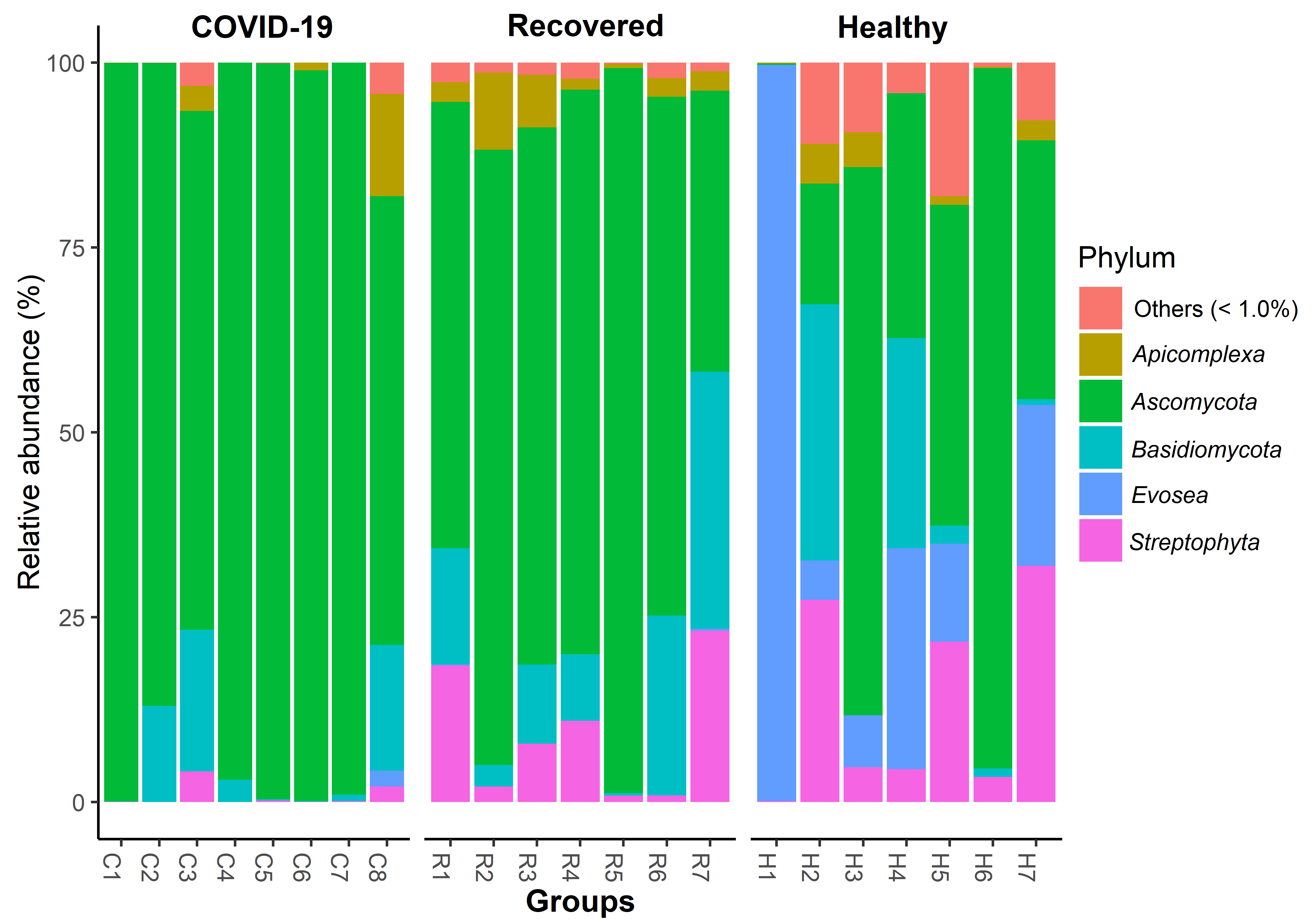

Supplement: S1 Fig — The phylum-level taxonomic profile of fungal microbiomes in COVID-19 (C1-C8), Recovered (R1-R7) and Healthy (H1-H7) nasopharyngeal samples. Phyla with > 1% mean relative abundance are represented by different color codes against respective sample groups. Others (< 1%) indicates the rare taxa in each group, with mean relative abundance of < 1%. (PNG) [file pone.0278134.s001.png]

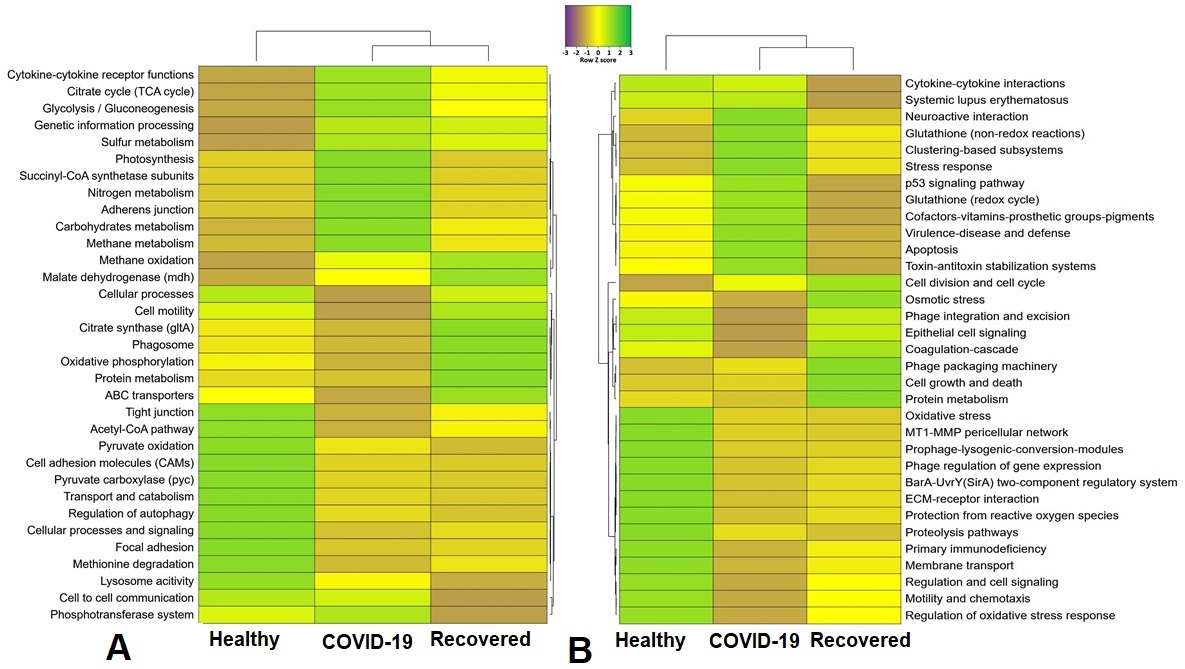

Supplement: S2 Fig — Heatmap showing (A) KEGG orthologues (KOs) and (B) SEED subsystems associated with fungal metabolism in Healthy, COVID-19 and Recovered metagenomes. (JPG) [file pone.0278134.s002.jpg]
