# Supplementary material for: Transcriptome analysis reveals increased abundance and diversity of opportunistic fungal pathogens in nasopharyngeal tract of COVID-19 patients
Source: PLoS One. 2023 Jan 19;18(1):e0278134. doi: 10.1371/journal.pone.0278134 (PMC9851516; doi:10.1371/journal.pone.0278134)
Supplement: S1 Table — (DOCX) [file pone.0278134.s003.docx]

**Table S1.** Demographic characteristics of the study people: clinical diagnosis, treatment and recovery history of SARS-CoV-2 infections.

| **Sample_ID** | **Age** | **Gender** | **COVID-19 symptoms** | **COVID-19 diagnosis**  **(Days after onset of symptoms)** | **COVID-19 medicine** | **Recovered***  **(Days after COVID-19 diagnosis)** |
| --- | --- | --- | --- | --- | --- | --- |
| COVID-1 | 22 | M | Yes | 3 | 14 | 14 |
| COVID-2 | 35 | F | Yes | 5 | 12 | 12 |
| COVID-3 | 45 | M | Asymptomatic | NA | 11 | 11 |
| COVID-4 | 39 | M | Yes | 5 | 14 | 14 |
| COVID-5 | 32 | M | Yes | 5 | 21 | 21 |
| COVID-6 | 34 | M | Yes | 9 | 21 | 21 |
| COVID-7 | 38 | F | Yes | 4 | 14 | 14 |
| COVID-8 (ICU death) | 72 | M | Yes | 4 | 10 | NA |
| RECOV-1 | 38 | F | Asymptomatic | NA | 14 | 14 |
| RECOV-2 | 34 | M | No | 9 | 18 | NA |
| RECOV-3 | 22 | M | No | 5 | 17 | NA |
| RECOV-4 | 35 | F | Asymptomatic | NA | 12 | NA |
| RECOV-5 | 45 | M | No | 7 | 17 | NA |
| RECOV-6 | 32 | M | Asymptomatic | NA | 21 | NA |
| RECOV-7 | 39 | M | No | 4 | 14 | NA |
| Healthy-1 | 42 | M | No | NA | NA | NA |
| Healthy-2 | 48 | F | No | NA | NA | NA |
| Healthy-3 | 32 | M | No | NA | NA | NA |
| Healthy-4 | 66 | M | No | NA | NA | NA |
| Healthy-5 | 57 | F | No | NA | NA | NA |
| Healthy-6 | 48 | F | No | NA | NA | NA |
| Healthy-7 | 44 | M | No | NA | NA | NA |
| **Average** | **40.86** | **M = 68.18%, F = 31.82%** |  | **5.45** | **15.33** | **15.12** |

* The Recovered people became COVID-19 tests negative. NA: not applicable.
