# Supplementary material for: Transcriptome analysis reveals increased abundance and diversity of opportunistic fungal pathogens in nasopharyngeal tract of COVID-19 patients
Source: PLoS One. 2023 Jan 19;18(1):e0278134. doi: 10.1371/journal.pone.0278134 (PMC9851516; doi:10.1371/journal.pone.0278134)
Supplement: S2 Table — (DOCX) [file pone.0278134.s004.docx]

| **Fungal species** | **Relative abundances in the metagenomes of** | | |
| --- | --- | --- | --- |
|  | **Healthy** | **COVID-19** | **Recovered** |
| *Nannochloropsis oceanica* | 47.925 | 0.0004 | 0.0055 |
| *Saccharomyces pastorianus* | 34.416 | 0.4304 | 0.0329 |
| *Saccharomyces cerevisiae* | 2.797 | 88.6194 | 4.6598 |
| *Aspergillus pseudoglaucus* | 1.843 | 0.1676 | 4.4189 |
| *Aspergillus penicillioides* | 1.246 | 0.0018 | 36.6338 |
| *Paecilomyces variotii* | 1.240 | 0.370 | 1.080 |
| *Eremothecium gossypii* | 1.061 | 0.0427 | 1.2047 |
| *Auricularia polytricha* | 0.663 | -- | 0.0411 |
| *Arisaema ringens* | 0.411 | -- | -- |
| *Auricularia cornea* | 0.398 | 0.0001 | 0.0219 |
| *Malassezia restricta* | 0.398 | 0.0915 | 2.6311 |
| *Aspergillus glaucus* | 0.345 | 0.0018 | 1.1650 |
| *Aspergillus flavus* | 0.331 | 0.0036 | 1.4428 |
| *Acanthamoeba castellanii* | 0.292 | -- | -- |
| *Malassezia globosa* | 0.265 | 0.0367 | 0.7296 |
| *Monosiga brevicollis* | 0.265 | -- | -- |
| *Aspergillus fumigatus* | 0.252 | 0.0003 | 0.3368 |
| *Nelumbo nucifera* | 0.239 | -- | -- |
| *Eichleriella tenuicula* | 0.225 | -- | -- |
| *Candida glucosophila* | 0.212 | 0.0002 | -- |
| *Auricularia auricula-judae* | 0.212 | 0.0001 | 0.0027 |
| *Priapulus caudatus* | 0.186 | -- | -- |
| *Aspergillus oryzae* | 0.159 | 0.0025 | 10.0520 |
| *Rhizoctonia solani* | 0.159 | 0.0001 | -- |
| *Penicillium rubens* | 0.133 | 0.0004 | 0.1396 |
| *Spirometra erinaceieuropaei* | 0.133 | 0.0040 | -- |
| *Aspergillus niger* | 0.106 | 0.0002 | 0.2313 |
| *Vermamoeba vermiformis* | 0.053 | -- | 0.1971 |
| *Torulaspora delbrueckii* | 0.027 | 0.0053 | 0.0068 |
| *Pyricularia oryzae* | 0.027 | 0.0005 | 0.1985 |
| *Sydowia polyspora* | 0.027 | 0.0044 | 0.0438 |
| *Aspergillus nidulans* | 0.013 | 0.0052 | 0.0849 |
| *Aspergillus restrictus* | 0.013 | 0.0002 | 0.4449 |
| *Aspergillus magnivesiculatus* | -- | -- | 0.513 |
| *Aspergillus keveii* | -- | -- | 25.3634 |
| *Saccharomyces paradoxus* | -- | 0.0051 | 0.0246 |
| *Saccharomyces kudriavzevii* | -- | 0.0050 | 0.0110 |
| *Kluyveromyces lactis* | -- | 0.0322 | 0.000 |
| *Kazachstania aquatica* | -- | 0.0031 | -- |
| *Yarrowia lipolytica* | -- | 0.0040 | -- |
| *Colletotrichum gloeosporioides* | -- | 0.0140 | 0.0274 |
| *Parengyodontium album* | -- | 0.0041 | -- |
| *Cladosporium sphaerospermum* | -- | 0.0048 | -- |
| *Flavodon flavus* | -- | 0.0139 | -- |
| *Pseudozyma hubeiensis* | -- | -- | 0.2300 |
| *Phaffia rhodozyma* | -- | 10.2948 | -- |
| *Tarsonemidae gen. sp. AD1063* | -- | 0.0085 | 0.0575 |
| *Caenorhabditis remanei* | -- | 0.0048 | -- |
| *Trillium govanianum* | -- | -- | 0.5476 |
| *Guttulinopsis rogosa* | -- | 0.0080 | -- |

**Table S2:** Dysbiosis of mycobiomes after SARS-CoV-2 infections.
