# Supplementary material for: Transcriptome analysis reveals increased abundance and diversity of opportunistic fungal pathogens in nasopharyngeal tract of COVID-19 patients
Source: PLoS One. 2023 Jan 19;18(1):e0278134. doi: 10.1371/journal.pone.0278134 (PMC9851516; doi:10.1371/journal.pone.0278134)
Supplement: S3 Table — (DOCX) [file pone.0278134.s005.docx]

**Table S3** Metabolic functional potentials of the fungal microbiome.

| **KEGG Pathways** | | | |
| --- | --- | --- | --- |
|  |  |  |  |
| **KO's** | **Healthy** | **COVID-19** | **Recovered** |
| Cytokine-cytokine receptor functions | 4.40 | 17.94 | 12.48 |
| Genetic information processing | 6.43 | 12.62 | 11.90 |
| Nitrogen metabolism | 1.56 | 8.59 | 2.18 |
| Sulfur metabolism | 0.47 | 5.59 | 4.36 |
| Carbohydrates metabolism | 0.81 | 5.24 | 2.29 |
| Methane oxidation | 1.43 | 4.60 | 6.14 |
| mdh; malate dehydrogenase | 1.36 | 4.60 | 7.88 |
| Photosynthesis | 1.15 | 4.05 | 1.02 |
| Glycolysis / Gluconeogenesis | 2.40 | 3.95 | 3.19 |
| Succinyl-CoA synthetase subunits (sucC, sucD) | 1.84 | 3.95 | 1.88 |
| Pyruvate oxidation | 7.95 | 3.77 | 2.31 |
| Methane metabolism | 0.84 | 3.37 | 1.49 |
| Citrate cycle (TCA cycle) | 1.90 | 2.50 | 2.26 |
| Lysosome acitivity | 3.74 | 1.92 | 0.51 |
| Cell adhesion molecules (CAMs) | 3.22 | 1.80 | 1.72 |
| PC, pyc; pyruvate carboxylase | 5.53 | 1.65 | 1.37 |
| Cell to cell communication | 1.79 | 1.62 | 0.63 |
| Transport and catabolism | 3.76 | 1.52 | 1.35 |
| Phosphotransferase system | 1.29 | 1.50 | 0.54 |
| CS, gltA; citrate synthase | 2.52 | 1.34 | 5.43 |
| Phagosome | 1.25 | 1.07 | 1.72 |
| Adherens junction | 0.18 | 0.91 | 0.24 |
| Cellular processes | 12.37 | 0.91 | 10.84 |
| Cellular processes and signaling | 14.91 | 0.80 | 3.07 |
| Tight junction | 3.22 | 0.78 | 1.72 |
| Oxidative phosphorylation | 1.92 | 0.66 | 3.99 |
| Regulation of autophagy | 1.15 | 0.61 | 0.53 |
| Cell motility | 1.32 | 0.57 | 1.54 |
| Focal adhesion | 3.22 | 0.50 | 0.73 |
| Methionine degradation | 2.81 | 0.42 | 1.02 |
| ABC transporters | 0.32 | 0.24 | 0.40 |
| Acetyl-CoA pathway | 2.49 | 0.20 | 1.11 |
| Protein metabolism | 0.47 | 0.19 | 2.16 |
|  |  |  |  |
| **SEED Subsystems** | | | |
|  |  |  |  |
| **SEED Functions** | **Healthy** | **COVID-19** | **Recovered** |
| Cytokine-cytokine interactions | 6.96 | 6.34 | 2.18 |
| Cell division and cell cycle | 1.78 | 4.60 | 6.14 |
| Neuroactive interaction | 1.78 | 4.60 | 1.54 |
| Glutathione:_non-redox_reactions | 1.78 | 4.60 | 2.60 |
| Clustering-based subsystems | 2.98 | 3.95 | 3.19 |
| Stress response | 2.98 | 3.95 | 3.19 |
| p53 signaling pathway | 2.98 | 3.95 | 1.69 |
| Glutathione:_redox_cycle | 2.98 | 3.95 | 1.69 |
| Cofactors, vitamins, prosthetic groups, pigments | 2.98 | 3.95 | 1.69 |
| Phage_integration_and_excision | 3.18 | 2.64 | 3.18 |
| Virulence, disease and defense | 2.36 | 2.50 | 2.26 |
| Apoptosis | 2.36 | 2.50 | 2.26 |
| Systemic lupus erythematosus | 2.36 | 2.50 | 0.46 |
| Toxin-antitoxin stabilization systems | 1.13 | 1.73 | 0.60 |
| Oxidative stress | 7.98 | 1.65 | 1.37 |
| MT1-MMP pericellular network | 8.71 | 1.65 | 1.37 |
| Proteolysis pathways | 3.13 | 1.35 | 0.96 |
| ECM-receptor interaction | 3.13 | 1.34 | 1.65 |
| Osmotic stress | 3.13 | 1.34 | 5.43 |
| Primary immunodeficiency | 3.13 | 1.34 | 1.99 |
| Prophage_lysogenic_conversion_modules | 3.13 | 0.98 | 1.04 |
| Epithelial cell signaling | 1.78 | 0.98 | 1.76 |
| Motility and chemotaxis | 3.02 | 0.95 | 2.01 |
| Regulation and cell signaling | 3.02 | 0.94 | 1.94 |
| Coagulation_cascade | 2.13 | 0.86 | 2.65 |
| Phage_packaging_machinery | 0.29 | 0.86 | 3.19 |
| Regulation_of_oxidative_stress_response | 3.18 | 0.78 | 1.99 |
| Membrane transport | 2.40 | 0.60 | 1.22 |
| Phage_regulation_of_gene_expression | 3.18 | 0.36 | 0.71 |
| Cell growth and death | 0.18 | 0.23 | 0.84 |
| BarA-UvrY(SirA) two-component regulatory system | 7.74 | 0.12 | 1.19 |
| Protection_from_reactive_oxygen_species | 2.13 | 0.11 | 0.46 |

**Data S1** Please see the attached Excel file.
